# Shoulder motor performance assessment in the sagittal plane in children with hemiplegia during single joint pointing tasks

**DOI:** 10.1186/1475-925X-13-106

**Published:** 2014-07-29

**Authors:** Domenico Formica, Maurizio Petrarca, Stefano Rossi, Loredana Zollo, Eugenio Guglielmelli, Paolo Cappa

**Affiliations:** 1 Laboratory of Biomedical Robotics and Biomicrosystems, Università Campus Bio-Medico di Roma, via Alvaro del Portillo 21-00128, Rome, Italy; 2 MARLab Movement Analysis and Robotics Laboratory - Neuroscience and Neurorehabilitation Department, “Bambino Gesù” Children’s Hospital, Via Torre di Palidoro, 00050 Passoscuro (Fiumicino), Rome, Italy; 3 DEIM Department of Economics and Management – Industrial Engineering, University of Tuscia, Via del Paradiso 47, 01100 Viterbo, Italy; 4 Department of Mechanical and Aerospace Engineering, “Sapienza” University of Rome, Via Eudossiana, 18-00184, Rome, Italy

**Keywords:** Motor assessment, Hemiplegia, Cerebral palsy, Kinematics, Biomechanics

## Abstract

**Background:**

Pointing is a motor task extensively used during daily life activities and it requires complex visuo-motor transformation to select the appropriate movement strategy. The study of invariant characteristics of human movements has led to several theories on how the brain solves the redundancy problem, but the application of these theories on children affected by hemiplegia is limited. This study aims at giving a quantitative assessment of the shoulder motor behaviour in children with hemiplegia during pointing tasks.

**Methods:**

Eight children with hemiplegia were involved in the study and were asked to perform movements on the sagittal plane with both arms, at low and high speed. Subject movements were recorded using an optoelectronic system; a 4-DOF model of children arm has been developed to calculate kinematic and dynamic variables. A set of evaluation indexes has been extracted in order to quantitatively assess whether and how children modify their motor control strategies when perform movements with the more affected or less affected arm.

**Results:**

In low speed movements, no differences can be seen in terms of movement duration and peak velocity between the More Affected arm (MA) and the Less Affected arm (LA), as well as in the main characteristics of movement kinematics and dynamics. As regards fast movements, remarkable differences in terms of strategies of motor control can be observed: while movements with LA did not show any significant difference in Dimensionless Jerk Index (JI) and Dimensionless Torque-change Cost index (TC) between the elevation and lowering phases, suggesting that motor control optimization is similar for movements performed with or against gravity, movements with MA showed a statistically significant increase of both JI and TC during lowering phase.

**Conclusions:**

Results suggest the presence of a different control strategy for fast movements in particular during lowering phase. Results suggest that motor control is not able to optimize Jerk and Torque-change cost functions in the same way when controls the two arms, suggesting that children with hemiplegia do not actively control MA lowering fast movements, in order to take advantage of the passive inertial body properties, rather than to attempt its optimal control.

## Introduction

Pointing is a motor task extensively used during daily life activities to indicate, to ask for, to emphasize, to share situations, concepts, emotions, and more. Executing a pointing task requires the mental representation of a spatial reference frame that could be egocentric and/or allocentric. Pointing at an object positioned far from the body involves both mechanisms in humans from the age of 7-8-years-old, while younger children represent objects in space using mainly egocentric cues [1]. When visual feedback is available, the spatial frame of reference is object-centered and requires the involvement of both mechanisms [1].

The arm movement requires visuo-spatial transformation and cognitive processes. These processes select the appropriate movement strategy to allow the functional precision needed to successfully plan, execute and control the target task, among the large number of possible solutions offered by a multi-link, multiple degree of freedom (DOF) system (known as “the redundancy problem” [2]). The control of the different DOFs integrates the constraints imposed by body structure, the environmental context and the specific selected task [3]; in fact, both movement speed and direction affect motor control. More precisely, as regards movement speed, rapid movements involve the balancing of inertial and centrifugal forces while slow movements mainly have to balance the gravitational force [4]. As for movement direction, horizontal movement is executed on a gravitational equi-potential plane while during vertical movement potential energy varies.

The study of invariant characteristics of human movements under different conditions (speed, amplitude or direction of movements, and externally applied forces) has led to several theories on which variables the Central Nervous System (CNS) uses to solve the redundancy problem about the control of multiple DOFs. Most popular theories can be classified in: (i) *optimization-based approaches*, which propose minimum principle where a global cost function is minimized by the CNS. To this class belong, among others, minimum acceleration [5], minimum-effort [6], minimun-jerk [7], minimum-torque change [8], minimum-variance [9], uncontrolled manifold (UCM) [10], optimal feedback control [11]; (ii) *phenomenological approaches*, which seek invariants of motion variables to propose empirical laws. Popular empirical laws are Fitt’s law [12], Donders’/Listing’s law [13,14], isochrony [15], linearly related joint velocities [16], ‘two-third power law’ [17].

While over the past few years several studies have focused on the movement analysis and motion control characteristics in healthy subjects, the application of the above mentioned theories to clinical practice on children affected by hemiplegia is limited [18,19]. In children with hemiplegia the control mechanism on reciprocal and co-contraction muscle activities is disrupted, because it is mainly exerted by the control on the stretch reflex threshold, that is in turn influenced by central and peripheral mechanisms [18]. Objective and quantitative assessment of neuro-motor deficits is of paramount importance in clinical practice for choosing appropriate rehabilitation treatment, monitoring the recovery progress, and using this information to adjust the therapy.

Though the study does not propose a new diagnostic tool, its main purpose is to provide a reliable, quantitative evaluation of the upper limb/shoulder motor strategies in order to assess whether and how children modify their motor control strategies when perform movements with the more affected or less affected arm, during single joint pointing task performed in a standing posture.

## Materials and methods

### Subjects

Eight children with hemiplegia (four male and four female) were involved in this study (mean age 9.6 ± 2.7 (SD) years; age range from 6 to 14 years); they were recruited from the Paediatric Neuro-Rehabilitation Division of the Children’s Hospital “Bambino Gesù”. The main characteristics of the subjects involved in the experimentation are summarized in Table 1.

**Table 1:** Overview of the main characteristics of the hemiplegic children involved in the study

**Subject**	**Age**	**Body mass [kg]**	**Height [cm]**	**Affected arm**	**Gender**
#1	14	55	177	Right	Male
#2	12	36.5	144	Right	Male
#3	6	22.5	115	Left	Male
#4	8	35.6	129	Right	Male
#5	8	26.5	127	Right	Female
#6	9	28	135	Left	Female
#7	12	41	149	Right	Female
#8	8	23.6	120	Left	Female

The subjects who participated in the study met the following inclusion criteria: children with congenital hemiplegia and ability in pointing task. Exclusion criteria were presence of: seizures, arousal and cognitive problem, and severe muscle contracture. Standard functional and neurological assessment was administered.

The purpose and the experimental methodologies used in the study were fully explained to the participants. All subjects and their parents provided informed consent to be involved in the study. The protocol was approved by the Research Ethics Board of the Children’s Hospital “Bambino Gesù” of Rome.

### Experimental setup

Kinematic data were collected using an optoelectronic system equipped with six cameras set at a sampling rate of 120 images per second (Vicon 512, Oxford Metrics, UK). Prior to each experiment, the system was statically and dynamically calibrated so that the overall RMS error of marker coordinates in three-dimensional space was less than 1 mm.

We used a subset of the PlugInGait protocol: twelve spherical retro-reflective markers (14 mm in diameter) were placed on anatomical landmarks on the upper extremities: one on the coracoid process; one on the center of the elbow joint; one on the line connecting the coracoid process and the elbow joint; two on the wrist; and, finally, one on the third metacarpal ray. The same skilled therapist placed the markers in all trials.

### Experimental protocol

Patients stood bilaterally in a comfortable upright position in the center of a large room (12 × 5 m^2^) of the Movement Analysis and Robotics Laboratory of the Children’s Hospital “Bambino Gesù” (MARLab). Their feet were placed symmetrically with respect to the subject’s vertical axis and were slightly less than shoulder width apart; their arms hung vertically and comfortably at their sides. The experimenter instructed the patients to perform the pointing task asking them to: (a) point with the index finger to a stationary object placed on a wall surface about four meters in front of them at shoulder level; and (b) return from the elevation to the initial rest position. The two simple movements conducted in the elevation and the lowering phases must be individually performed without stopping. The subject was also instructed to do not flex the elbow and no other instructions were given by the experimenter on the path that the patient has to follow. We decided to discard trials where the patients flexed the elbow significantly (>10 deg), as revealed by movement analysis system, or took a step to maintain equilibrium.

We chose a single joint vertical arm movement because: (a) it had been previously proposed in literature [4]; (b) it is controlled separately by each hemisphere, thus comparison between more and less affected side can be made [20]. We chose a standing posture because: (a) it represents a natural condition for far objects pointing tasks and (b) it does not impose other constraints in upper limb movements.

The experimental analysis included two speed conditions: (a) low speed (L), i.e. the patient has to replicate the movement model offered by the experimenter (the time length during the elevation and lowering was about 1.5 s each); (b) high speed (H), i.e. the patient has to move the arm as quick as possible. Then, the participant has to repeat the previously indicated conditions with both the less and the more affected arm.

Therefore, the entire protocol consisted of four series of trials for each patient: L-LA, H-LA and L-MA, H-MA for low (L) and high (H) speed trials with both the more affected (MA) and less affected (LA) arm, respectively. Each of the previously mentioned four conditions was repeated nine times, in a random order, so a total of thirty-six trials were performed by each patient. The total number of trials represents a reasonable trade-off between the reliability of 3D data made available by motion analysis system [21,22] and the need to avoid subjects reporting fatigue at the end of the assessment session. For each subject, the entire experimental session lasted approximately thirty minutes; all the subjects completed the entire session.

### Data analysis

Reflective markers were manually identified using Vicon Workstation and all marker trajectories were filtered using a Woltring filter (size 30). In-house developed Matlab (release VII, Mathworks, USA) software packages were used for data processing.

The coordinates of the centres of rotation (COR) of shoulder, elbow and wrist joints (COR_SH_, COR_EL_ and COR_WR_, respectively) provided by the motion system were used as inputs for data processing and for extracting all the selected variables; then, the value of velocity and acceleration relative to the above mentioned CORs were numerically determined. After a visual inspection of each single trial, movement onset was defined as the time instant in which the linear tangential velocity of the COR_WR_ exceeded of a velocity threshold, while the end of the entire trial was defined as the time instant in which velocity dropped below the same threshold. We defined two absolute thresholds, one for slow movements and the other for fast movements: the two thresholds are set as the 5% of the mean wrist peak velocity averaged over all subjects in the two speed conditions (equal to 0.063 m/s and 0.172 m/s for slow and fast movements respectively).

Each trial was decomposed into two subsets: (a) the elevation phase, that is performed against gravity, and (b) the lowering phase. The temporal separation of the two sub-movements was detected by the time instant where the vertical component of the wrist velocity changed its sign.

**Figure 1:**
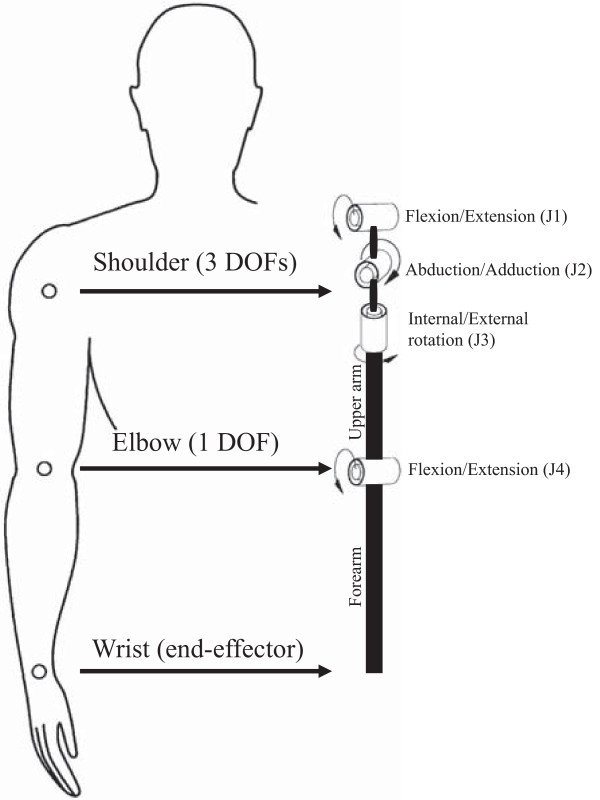
**Kinematics of the proposed model.** The kinematic diagram of the proposed biomechanical model and the sequence in which the different DOFs are defined. For each DOF arrows represent the direction of positive angles and torques (right arm). To compare right and left movements, the positive direction of J2 and J3 DOFs is opposite to the one showed in figure.

A kinematic and dynamic model of the upper limb was developed taking into account of two opposite requirements: to reproduce in adequate way arm behaviour during the pointing tasks and to simplify the mathematical complexity of the complete model of human arm based of the seven DOF scheme. The developed model allows extracting, from the COR positions, a complete characterization of the limb motion in terms of kinematic variables (angles, velocities and accelerations of arm joints), dynamic variables (joint torques) and energetic variables (potential and kinetic energy and powers related to each joint). The model used in the present work consists of a 4-DOF kinematic chain with four revolute joints and two rigid bodies. The four revolute joints simulate the three shoulder DOFs (ordered in the following sequence: flexion/extension, abduction/adduction, and internal/external rotation) and one DOF of the elbow (flexion/extension). The two links, representing the upper arm and forearm, connect the COR_SH_ to the COR_EL_ and the COR_EL_ to the COR_WR_, respectively. Figure 1 shows the kinematic chain of the proposed model and the sequence in which the different DOFs are defined.

The following assumptions were made: (a) body segments were supposed completely rigid; (b) tissues properties were neglected; (c) link centres of gravity were supposed located in a fixed point; and, finally, (d) joints were considered frictionless. The model does not take into account forearm pronation/supination and wrist joint movements because the collected data showed a negligible contribution, thus the COR_WR_ was considered as the end point of the model.

The lengths of upper arm and forearm were calculated as the distances between two subsequent CORs, while mass, inertia and centres of mass of the links were estimated for each subject by resorting to a database of average anthropometric data [23].

The dynamics of the arm can be represented by the following equation:

(1) $$B\left(q\right)\ddot{q}+C\left(q,\dot{q}\right)\dot{q}+G\left(q\right)=T$$

where:

$q,\dot{q},\ddot{q}\in R^{4x1}$ are the arm joint position, velocity and acceleration vectors, respectively;

*B*(*q*) ∈ *R*^4*x*4^ is the kinetic energy matrix in the joint space;

$C\left(q,\dot{q}\right)\dot{q}\in R^{4x1}$ is the vector of centrifugal and Coriolis torques;

*G*(*q*) ∈ *R*^4*x*1^ is the vector of gravitational torques;

*T* ∈ *R*^4*x*1^ is the vector of torques exerted by joint muscles.

The developed 3D model uses COR_SH_, COR_EL_ and COR_WR_ as inputs and provides as outcomes the following three sets of variables: kinematic variables, i.e. angular positions, velocities and accelerations for each DOF of the model (vectors $q,\dot{q},\ddot{q}$ in eq. 1); dynamic variables, i.e. inertial, Coriolis and gravitational components of joint torques for each DOF of the model; energetic variables, i.e. system potential and kinetic energy, and powers related to each DOF of the model.

Model kinematics and dynamics has been implemented in Matlab (Mathworks, USA). The selected variables allowed the estimation of performance indexes aimed at measuring motor performance of the hemiplegic subjects, and quantitatively assessing for each subject the level of motor impairment of the MA with respect to the LA. The extracted set of indexes was mostly resumed from previous studies on human motor planning and execution of movements in healthy subjects [7,8,24]. They are reported in the following.

### Kinematic indexes

• Movement Duration (MD): defined as the time length necessary to the patient to perform the elevation and the lowering phases.

• Peak Velocity (PV): the maximum value of the velocity magnitude of the COR_wr_ during arm elevation and lowering.

• Time to Peak Velocity (TPV): defined as the percentage time with respect to MD necessary to the patient to reach the peak velocity. TPV characterizes the movement timing because gives information on the relation between acceleration and deceleration time in the elevation and the lowering phases. TPV assumes a value of 0.5 in planar (without gravity) point-to-point reaching movements [7], due to the fact that acceleration time length is identical to the deceleration one, while in the movements performed against gravity it can assume different values due to the different motion constraints [4,24,25].

• Peak to Mean velocity Ratio (PMR): defined as the ratio between PV and the mean velocity of the COR_wr_. This index synthesizes the shape of velocity profiles: for planar movements at minimum jerk (i.e. with maximum smoothness of the movements), it assumes a value of 1.875 [7] while it assumes different values for movements performed against gravity [24].

### Cost indexes

The cost indexes are based on cost functions used in neuro-scientific studies to model and to interpret how the brain plans and executes point-to-point movements. The basic assumption is that in healthy subjects the brain tries to optimize motion by minimizing cost functions related to kinematic or dynamic variables [26,27]. In the present paper, it is assumed that the same cost functions could be successfully used to detect and characterize pathological behaviours, in particular in the comparison between MA and LA, as done by previous works for different pathologies [28,29]. To this purpose, two indexes derived by the cost functions related to two popular motor control theories were taken into account.

#### Dimensionless Jerk Index (JI)

It derives by the minimum-jerk cost originally proposed by Hogan for single joint movements [30] and later successfully applied by Flash and Hogan [7] to model planar multijoint reaching movements. They stated that, during such movements, the brain tries to maximize movement smoothness, by minimizing the first derivative of Cartesian hand acceleration (i.e. jerk). Here we propose to use as evaluation index of movement smoothness the dimensionless jerk index introduce in a previous work by Takada and colleagues [29], defined as:

(2) $$\mathrm{JI}=\frac{T^{5}}{L^{2}}\int_{0}^{T}\left({\left(\frac{d^{3}x}{dt^{3}}\right)}^{2}+{\left(\frac{d^{3}y}{dt^{3}}\right)}^{2}+{\left(\frac{d^{3}z}{dt^{3}}\right)}^{2}\right)\mathrm{dt}$$

where *t* is movement time, that goes from 0 (movement onset) to T (movement duration), and x, y, z are the coordinates of the COR_WR_ at the generic time *t*. As the movements were carried out at different speeds among trials, index is normalized in order to allow appropriate comparisons, using the normalizing factor $\frac{T^{5}}{L^{2}}$, where L is the 3D pathway length of each movement. It can be noted that normalizing using $\frac{T^{5}}{L^{2}}$ is equivalent of using $\frac{T^{3}}{{\bar{v}}^{2}}$, where $\bar{v}$ the mean speed of the movement.

We chose this index because Hogan and Sternad showed that it can properly quantify common deviations from smooth, coordinated movement, without being affected by movement duration and velocity [31].

#### Dimensionless Torque-change Cost index (TC)

It derives by the torque-change cost function originally proposed by Uno et al. [8] as an alternative approach to the minimum-jerk theory. In contrast with the minimum jerk model, the minimum torque-change model proposes that planned trajectories depends on arm dynamics. According to the original definition by Uno and colleagues, and normalized similarly to the JI, the TC index is defined as:

(3) $$TC=T\int_{0}^{T}\left({\left(\frac{1}{{\bar{\tau }}_{J1}}\frac{d\tau_{J1}}{\mathrm{dt}}\right)}^{2}+{\left(\frac{1}{{\bar{\tau }}_{J2}}\frac{d\tau_{J2}}{\mathrm{dt}}\right)}^{2}+{\left(\frac{1}{{\bar{\tau }}_{J3}}\frac{d\tau_{J3}}{\mathrm{dt}}\right)}^{2}+{\left(\frac{1}{{\bar{\tau }}_{J4}}\frac{d\tau_{J4}}{\mathrm{dt}}\right)}^{2}\right)d\tau$$

where *τ*_*J*1_, *τ*_*J*2_, *τ*_*J*3_, *τ*_*J*4_ are commanded torques (i.e. the torques exerted by the muscles to generate the movement) calculated by the model for the four different DOFs (see Figure 1), ${\bar{\tau }}_{J1},{\bar{\tau }}_{J2},{\bar{\tau }}_{J3},{\bar{\tau }}_{J4}$ are the mean torques (mean values of the norm of each joint torque over the movement), and *t* is the time as defined in Eq. 2. Similarly to JI, TC is dimensionless in order to allow appropriate comparisons across different trials.

### Statistical analysis

For each subject the average values and the Standard Deviation (SD) were calculated for all the above mentioned indexes. Significant differences of the proposed indexes in the different conditions were evaluated using a series of two-way ANOVA tests (“used arm” x “movement direction”), using indexes as dependent variables. These tests were carried out for both low and high speed movements. Since the proposed indexes are strongly affected by large difference in movement velocity, and its effect on index values is easily predictable, we grouped data for movement velocity, separating L movements from H ones, in order to increase the power of the post-hoc tests for multiple comparisons. For all the performed tests the statistical significance was accepted when p < 0.05. For the ANOVA analyses, the differences among the different groups have been assessed using Dunn-Sidak correction for post-hoc tests.

## Results

**Figure 2:**
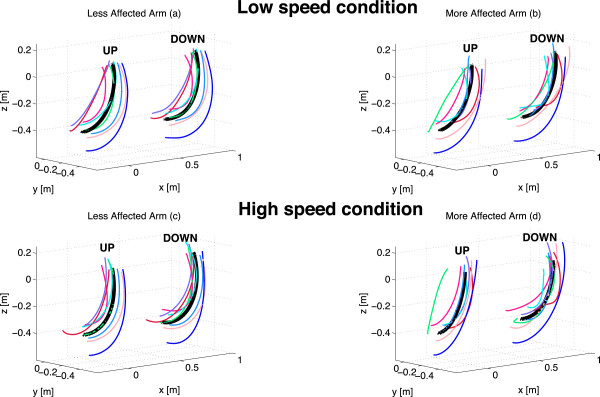
**Mean wrist trajectories.** Average wrist trajectories for each subject (coloured lines) together with the overall mean trajectories averaged over all subjects (black thick lines), gathered in L-LA **(a)**, L-MA **(b)**, H-LA **(c)** and H-MA **(d)** conditions. The centre of rotation of shoulder is the origin of the reference frame. Separate trajectories are represented for the elevation (UP) and lowering phase (DOWN), with the DOWN trajectories notionally shifted of 0.5 m on the x-axis, in order to distinguish them from the UP ones.

**Figure 3:**
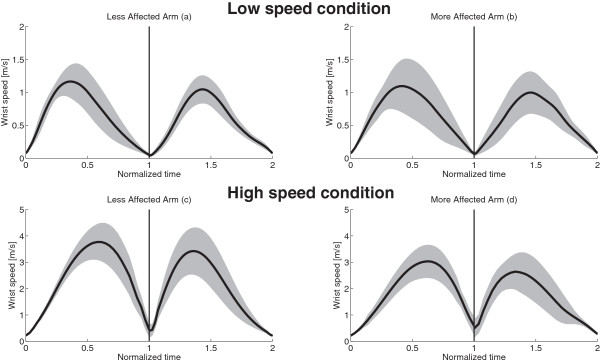
**Mean wrist speed.** Mean wrist velocity magnitude as a function of time, averaged over all subjects (black lines and grey shadows represent mean ± SD): values gathered in L-LA **(a)**, L-MA **(b)**, H-LA **(c)** and H-MA **(d)** conditions. The normalized time goes from 0 to 1 for the elevation phase and from 1 to 2 for the lowering one.

**Figure 4:**
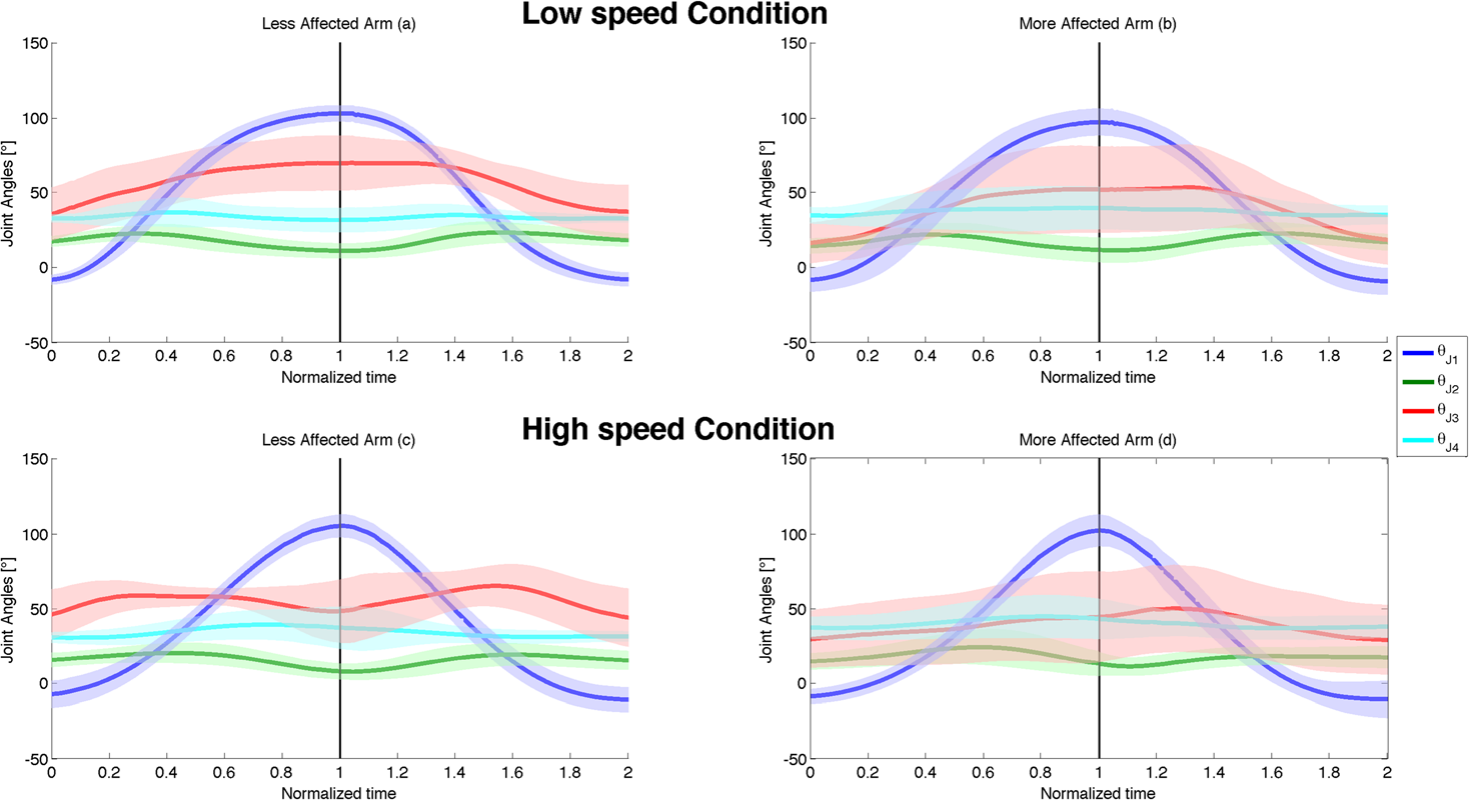
**Shoulder total power.** Shoulder total power as a function of time, averaged over all subjects (black lines and grey shadows represent mean ± SD): values gathered in L-LA **(a)**, L-MA, **(b)**, H-LA **(c)** and H-MA **(d)** conditions. The normalized time goes from 0 to 1 for the elevation phase and from 1 to 2 for the lowering one.

Average wrist trajectories for each subject together with the overall mean trajectories, averaged over all subjects, are represented in Figure 2, for both speed conditions (L and H) and used arms (LA and MA). Figure 3 shows the time-normalized profile of wrist velocity magnitude, averaged over all the subjects (mean ± SD), for the two speed conditions (L and H conditions) performed with both arms (LA and MA). While exhibiting a quite relevant inter-subjects variability, kinematic characteristics of wrist movements (both trajectories and velocities) show that mean paths are similar for L-LA compered to L-MA; however, at low speed, wrist velocity exhibits a higher variability for MA movements with respect to LA ones. Such difference cannot be seen in H condition (Figure 3c and Figure 3d), although pathological movements are characterized by lower velocity values (both peak and mean velocity), as also shown by MD and PV indexes presented in the following. We also analysed the kinematic characteristics of children movements in joint space, by calculating joint angles in the four experimental conditions averaged over all the subjects (mean ± SD) (Figure 4): LA and MA show similar behaviours both in terms of range of motion and joint angle profiles; nevertheless, MA movements exhibit a higher than normal variability in the internal/external rotation (J3 DOF) of the shoulder with respect to LA (SD of J3 angle is from 50% to 100% higher in MA than in LA).

**Figure 5:**
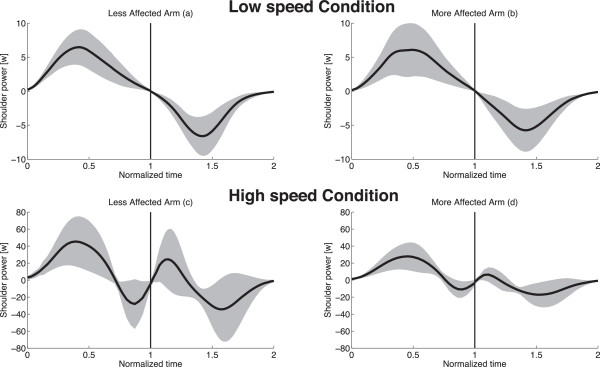
**Joint angles.** Joint angular displacements as a function of time, averaged over all subjects (solid lines and coloured shadows represent mean ± SD): values gathered in L-LA **(a)**, L-MA, **(b)**, H-LA **(c)** and H-MA **(d)** conditions. The normalized time goes from 0 to 1 for the elevation phase and from 1 to 2 for the lowering one. The four coloured curves represent the four DOFs of the kinematic model, as defined in Figure 1.

**Figure 6:**
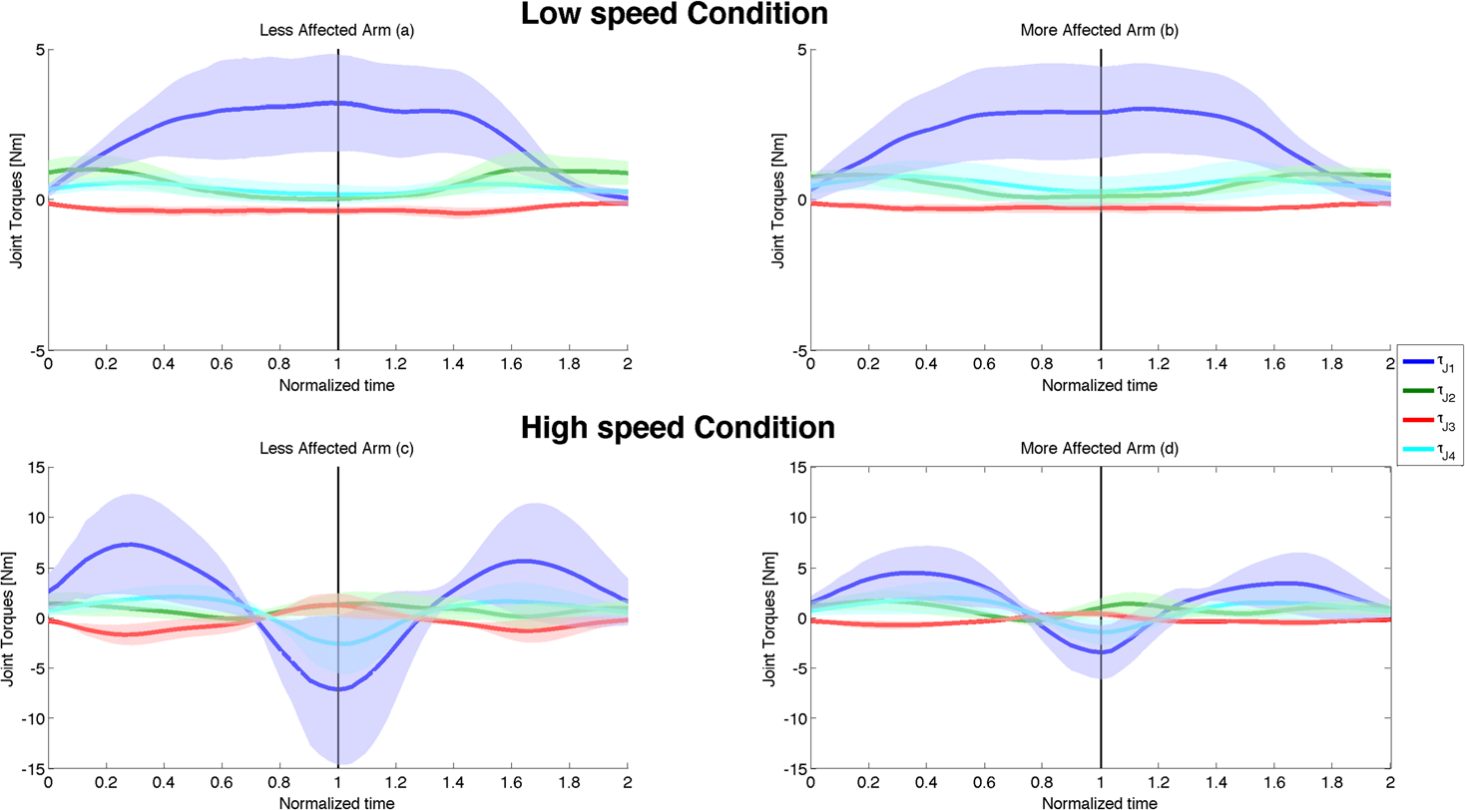
**Joint torques.** Joint torques as a function of time, averaged over all subjects (solid lines and coloured shadows represent mean ± SD): values gathered in L-LA **(a)** L-MA, **(b)**, H-LA **(c)** and H-MA **(d)** conditions. The normalized time goes from 0 to 1 for the elevation phase and from 1 to 2 for the lowering one. The four coloured curves represent the four DOFs of the kinematic model, as defined in Figure 1.

Despite the limited differences between LA and MA movements in terms of kinematics, relevant differences can be found by examining the dynamic characteristics of the movements; Figure 5 shows time-normalized plots of shoulder power averaged over all the subjects (mean ± SD): for L condition, LA (Figure 5a) and MA (Figure 5b) appears to be quite similar, with a negative peak in the lowering phase that is similar in shape and amplitude to the positive peak exhibited in the elevation phase; on the contrary, for H condition a comparison of power plots between LA and MA (Figure 5c and Figure 5d) shows strong differences especially during the first half of lowering movements, where shoulder total power exhibits in H-LA condition a relatively extended period with positive values (for about the 30% of the downward movement time) that, conversely, is very limited in H-MA condition. Such difference in controlling the first half of the lowering phase for high speed movements, can be seen also in the muscle joint torques (Figure 6), where the flexion/extension shoulder torque of LA shows a negative peak similar in amplitude to the positive peak exerted during the elevation phase, which is strongly reduced for the MA.

Values of the proposed indexes averaged over the eight subjects are reported in Table 2 for low speed condition and Table 3 for high speed condition. The four MD values collected in L condition are similar and no significant differences were found between arms, while MD is higher in lowering phase than in elevation one for both arms (p < 0.05 for LA, p < 0.01 for MA). In H condition, instead, the MD values exhibited statistical significant differences between H-LA and H-MA during lowering phase (p < 0.01). In addition, focusing always on H condition, it is also worth noting that for MA the lowering phase lasted longer than the corresponding elevation one (p < 0.01). The PV values showed a similar behaviour to the MD ones, due to the obvious intrinsic relationship; in fact, the mean values observed in H-MA are different from those relative to H-LA in the both phases of the movement (p < 0.05 for the elevation phase, p < 0.01 for the lowering phase). As regards the TPV index, L-MA movements result statistically different between elevation and lowering phase (p < 0.01); more interestingly, statistically reliable differences were found for the downward movements between the two arms (p < 0.01). In H condition, TPV exhibits strong differences between the elevation and the lowering phases for both arms (p < 0.01), while no differences can be noted for LA and MA. Analogously, the PMR index shows a significant difference between elevation and lowering phases for both LA (p < 0.01) and MA (p < 0.05) in H trials.

**Table 2:** Overall results for low speed condition grouped as means and SDs

		**Low speed condition (L)**			
		**Elevation phase**		**Lowering phase**	
		**Less affected arm (H-LA)**	**More affected arm (H-MA)**	**Less affected arm (H-LA)**	**More affected arm (H-MA)**
Evaluation indexes [Mean ± SD]	MD [s]	1.36^(b)^ ± 0.39	1.32^(b)^ ± 0.45	1.59^(b)^ ± 0.37	1.53^(b)^ ± 0.49
	PV [m/s]	1.36 ± 0.37	1.27 ± 0.39	1.24 ± 0.33	1.18 ± 0.35
	TPV	0.36 ± 0.10	0.37^(b)^ ± 0.13	0.41^(a)^ ± 0.10	0.47^(a,b)^ ± 0.12
	PMR	2.14 ± 0.51	2.02 ± 0.35	2.24 ± 0.51	2.15 ± 0.37
	JI	9584 ± 23106	8752 ± 19183	18285 ± 39763	14343 ± 28419
	TC	105 ± 92	114 ± 274	126 ± 177	97 ± 90

As regards the two-ways ANOVA tests, apex “^a^” indicates statistically significant differences for the “used arm” (LA versus MA) and “^b^” for “movement phase” (Elevation versus Lowering), obtained using Dunn-Sidak correction for post-hoc tests.

**Table 3:** Overall results for high speed condition grouped as means and SDs

		**High speed condition (H)**			
		**Elevation phase**		**Lowering phase**	
		**Less affected arm (H-LA)**	**More affected arm (H-MA)**	**Less affected arm (H-LA)**	**More affected arm (H-MA)**
Evaluation indexes [Mean ± SD]	MD [s]	0.41^(b)^ ± 0.10	0.47^(b)^ ± 0.16	0.52^(a,b)^ ± 0.11	0.62^(a,b)^ ± 0.20
	PV [m/s]	3.75^(a)^ ± 0.89	3.36^(a)^ ± 0.73	3.57^(a)^ ± 0.92	3.10^(a)^ ± 0.67
	TPV	0.58^(b)^ ± 0.08	0.59^(b)^ ± 0.09	0.38^(b)^ ± 0.09	0.37^(b)^ ± 0.10
	PMR	1.71^(b)^ ± 0.14	1.79^(b)^ ± 0.32	1.92^(b)^ ± 0.21	1.91^(b)^ ± 0.36
	JI	1105 ± 1325	2087^(b)^ ± 4093	2737 ± 2424	7298^(b)^ ± 23254
	TC	148 ± 81	205^(b)^ ± 170	247 ± 154	307^(b)^ ± 361

As regards the two-ways ANOVA tests, apex “^a^” indicates statistically significant differences for the “used arm” (LA versus MA) and “^b^” for “movement phase” (Elevation versus Lowering), obtained using Dunn-Sidak correction for post-hoc tests.

In L condition both cost indexes (JI and TC) do not differ between arms and movement directions; on the contrary, significant differences can be observed for both JI and TC during high speed trials: anova tests showed a significant effect of “used arm” on TC in H condition (p = 0.0293), and a non-significant effect, but close to significance, on JI (p = 0.0572); moreover, both indexes showed a statistically significant effect of “movement direction” (p < 0.05 for JI and p < 0.01 for TC), while the effect of the interaction “used arm” x “movement direction” is not statistically significant.

**Figure 7:**
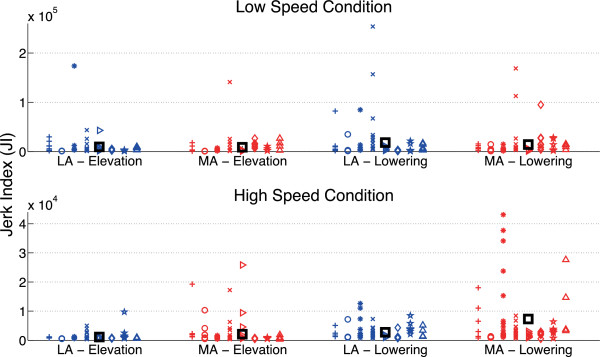
**Jerk Index values.** Scatterplot of Jerk Index (JI) values for individual subjects in the different testing conditions. Different marker shapes represent different subjects, while the black square markers represent the mean values for each testing condition (corresponding to values reported in Tables 2 and 3).

**Figure 8:**
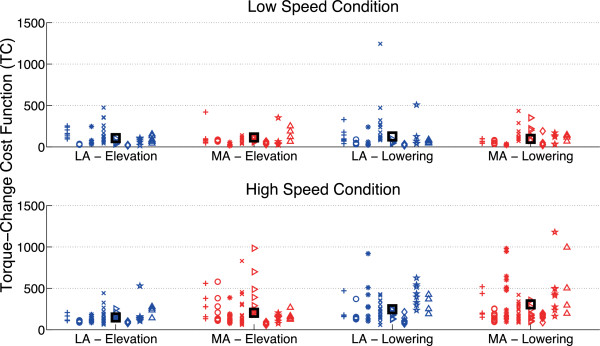
**Torque-Change Cost Index values.** Scatterplot of Torque - Change Cost Function (TC) values for individual subjects in the different testing conditions. Different marker shapes represent different subjects, while the black square markers represent the mean values for each testing condition (corresponding to values reported in Tables 2 and 3).

More interesting, the post-hoc tests showed that LA cost indexes do not exhibit differences between lowering and elevation phases, while MA lowering movements are characterized by statistically significant higher values of both cost indexes with respect to MA elevation movements (p < 0.05 for both JI and TC).The previously mentioned observation confirms that in H trials the most relevant differences between arms can be found mainly in lowering movements, where cost indexes, which aim at quantifying movement optimal behaviour, do not vary for LA while increase for MA. Scatterplots of JI and TC values for individual subjects are reported in Figures 7 and 8.

## Discussions

The analysis of kinematic characteristics of children movements did not show relevant differences between the two arms, in both low speed and high speed conditions; despite the fact that we did not ask to the subjects to point at a precise target, so to reduce the accuracy requirements, mean trajectories (Figure 2) and speed profile (Figure 3) are quite similar for the two arms, with the only statistically significant differences observable in high speed condition in terms of slower velocities and longer durations for MA movements (PV and MD indexes are statistically different for H-MA with respect to H-LA).

Global characteristics of velocity profiles such as timing (TPV index) and shape (PMR index) did not account for differences between arms except for the lowering phase in L condition. The temporal boundaries of the movement seem to be maintained despite the difficulty in controlling MA movements in an optimal way (see discussion below). Some differences can be noticed between upward and downward movements, in particular a tendency to increase TPV during lowering phase at slow velocity and the inverse at high velocity. Our results appear to be different from similar studies [24]. This discrepancy could be due to the different instructions given to subjects to perform tasks. Papaxantis and colleagues did not require execution accuracy but this request was implicit in the protocol, because the subjects were asked to move from one reference target to another, both of them close to the start and end trajectory points of the hand; on the contrary, in our task the subject moves the upper limb from the body to a target placed far from him and again downward near the trunk side. The range of motion of shoulder flexion-extension is around 90° in both studies but the movement constraints were different. The lower accuracy requested in our task induced our subjects to move the upper limb with high velocity during upward movement, as demonstrated by PV values, with a consequent more abrupt inversion of the movement; this could explain the higher TPV values in H condition during elevation movements and the lower TPV values in lowering ones. This hypothesis is reinforced by the observation that PV values in our tasks were opposite to the data reported in literature, i.e. they were higher in upward movements than in downwards ones [24]. Far target implied less control in upwards movements allowing higher velocity, that is, the movement boundaries include the system of constraints that implies both task instructions and physical limitations relative both to the body and to the environment.

Overall, the analysis of movement kinematics seems to suggest that the recruited hemiplegic children plan the execution of the movements in a similar way for the two arms, both in terms of end point position (wrist trajectory) and joint angles. Besides the obvious differences in MD and PV values, the only relevant note can be made regarding the higher variability of speed and joint angle profiles for L-MA; in particular SD of shoulder internal/external rotation angle is from 50% to 100% higher for MA with respect to LA in both speed condition, suggesting that some of the subjects are less able to perform a mono-axial movement with the MA (ideally a pure flexion/extension shoulder movements) and try to compensate by using other DOFs.

Most relevant differences can be appreciated analysing the dynamics of the movements, and considering the indexes related to optimization theories of motor control (JI and TC). The analysis of shoulder muscular power showed that differences can be seen in high speed condition between arms, especially in downward movements (Figure 5c and Figure 5d). Focusing the attention on movements with the LA (Figure 5c), we showed that in H condition shoulder overall power is positive (power generation) in the first half of both elevation and lowering movements, while negative (power absorption) during the second half in both movement directions. In fact, unlike movements in L conditions, where motor control is mainly aimed at balancing the arm’s weight, in H condition inertial and the centrifugal forces become much more relevant, and require to be compensated both during elevation and lowering phases [4]; differences in power generation during lowering H-MA movements suggest that children do not properly control the second part of the fast movements with MA, letting gravity and inertia forces to drive passively their arm.

This speculation seems to be confirmed by the analysis of the two cost indexes. As regards the dimensionless Jerk Index (JI), it has been purposely conceived [29] to properly quantify common deviations from smooth, coordinated movement, without being affected by movement duration and velocity [31]. Similarly the dimensionless Torque-change Cost index (TC) has been proposed to quantify the capability of motor control in optimizing torque-change cost function [8], without being influenced by differences in the commanded torques.As regards slow movements no differences can be seen for the two arms or for movement directions. This element is not surprising since during slow movements the effort of the control is devoted to compensate mainly the gravity, which is a postural force field, i.e. it is not influenced by movement dynamics but only by kinematics. Since we did not see differences in movement kinematics in L condition between LA and MA, we did not expect to find any significant difference in motor control behaviour. This is also demonstrated by strong similarities in both power (Figure 5a and 5b) and joint torque (Figure 6a and 6b) plots for L condition.

On the contrary, remarkable difference in terms of different strategies of motor control can be observed in high speed movements. During these movements, the motor control system has to accomplish the more demanding task of compensating for complex dynamics: in fact in this condition, motor system has not only to control desired trajectory against gravity and inertia, but also to balance interaction torques (i.e., those fictitious forces felt by one DOF due to the motion of the other DOFs), which has been shown to be particularly difficult task for patients with neuromotor impairments [32].

Results on the effect of “used arm” on the two cost indexes showed relevant difference in JI (albeit main effect of “used arm” is not statistically significant, p = 0.0572) and a statistically significant effect of “used arm” for TC index (p = 0.0293); moreover, both indexes showed a statistically significant effect of “movement direction” (p < 0.05 for JI and p < 0.01 for TC), while the effect of the interaction “used arm” x “movement direction” is not statistically significant.More interestingly, the main differences in the motor control behaviour have been shown by post-hoc tests when comparing differences between elevation and lowering phases for the two arms. While movements with LA do not show any significant difference in JI and TC during the two phases (suggesting that motor control optimization is similar for movements performed with or against gravity), movements with MA showed a statistically significant increase of both JI and TC during lowering phase. This result confirms the observation of difference in shoulder muscle power showed in Figures 5 and 6 during the first half of lowering fast movements. This suggests that the brain is not able to optimize Jerk Cost Function and Torque-change Cost Function in the same way when controls the two arms, making it plausible that the children with hemiplegia do not actively control MA lowering fast movements in order to take advantage of the passive inertial body properties rather than to attempt its active control.

## Conclusions

The main goal of this study is to assess differences in motor control strategies in children with hemiplegia. Our results show that the control of pointing movements depends on both movement velocity (slow or fast movements) and movement direction (upwards or downwards movements) as reported in literature. These differences are due to the different constraints imposed by gravitational forces exerted by gravity during slow speed trials and by inertial and interaction forces during high speed tasks.

In addition, the movement is also affected by context in which the task is conducted as evidenced by the difference in the results and experimental context between the present paper and previous reports. The more affected arm preserved the same general behaviour as the less affected one, but movements were affected by sensory-motor deficit. From this point of view a set of kinematic and kinetic indexes have been here proposed as the basis of a quantitative and objective assessment of upper-limb motor control in hemiplegic patients.

## Abbreviations

COR_SH_, COR_EL_, COR_WR_: Center of rotation of the shoulder, the elbow and the wrist; DOF: Degree of freedom; H: Trial performed at high speed; JI: Jerk Index; L: Trial performed at low speed; LA: Less Affected arm; MA: More Affected arm; MD: Movement Duration [s]; PMR: Peak to Mean velocity Ratio; PV: Peak Velocity [m/s]; SD: Standard Deviation; TC: Torque-change Cost function; TPV: Time to Peak Velocity.

## Competing interests

The authors declare that they have no competing interests.

## Authors’ contributions

MP conceived the study and performed experiments; DF defined the arm model and proposed the evaluation indices; DF and SR analysed data and performed the statistical analysis; DF, MP, SR, and LZ interpreted results of experiments; DF prepared figures; DF, MP and SR drafted manuscript; EG and PC coordinated the research; All authors edited and revised manuscript and approved final version.
